# Altered cerebellum functional network on newly diagnosed drug-naïve Parkinson’s disease patients with anxiety

**DOI:** 10.1515/tnsci-2020-0192

**Published:** 2021-10-29

**Authors:** Yirong Wang, Shushan Zhang, Haodi Yang, Xin Zhang, Shijia He, Jian Wang, Jian Li

**Affiliations:** Department of Neurology, Chengdu Second People’s Hospital, Chengdu, Sichuan Province, People’s Republic of China; Department of Neurology, Affiliated Hospital of North Sichuan Medical College, Nanchong, Sichuan Province, People’s Republic of China; North Sichuan Medical College, No. 234, Fujiang Street, Shunqing District, Nanchong, Sichuan Province, People’s Republic of China

**Keywords:** cerebellum, Parkinson’s disease, anxiety, functional connectivity, regional homogeneity

## Abstract

**Introduction:**

Damage to the cerebellar functional network may underlie anxiety symptoms in patients with Parkinson’s disease (PD). Herein we investigated the regional homogeneity (ReHo) and functional connectivity (FC) patterns of cerebellar and clinical correlates in PD patients with anxiety and explored their clinical significance.

**Methods:**

We enrolled 50 newly diagnosed drug-naïve PD patients and 30 normal controls (NCs). Twenty-six PD patients with anxiety symptoms (PD-A) and 24 PD patients without anxiety symptoms (PD-NA) were sorted into groups based on the Hamilton Anxiety Scale (HAMA). All included participants underwent rest-state functional magnetic resonance imaging (rs-fMRI) scanning. Cerebellar FC based on the seed-based method was used to investigate regional and whole brain function in PD-A, PD-NA, and NCs, and the relationship between the abnormal brain function and anxiety symptoms in PD patients was also detected.

**Results:**

Compared with the PD-NA group and the NCs, the ReHo value of the PD-A group was significantly decreased in the left medial frontal gyrus and increased in the left cerebellum. Further, left-cerebellum-based FC patterns were used to detect the decreased FC in the right cerebellum, while FC was increased in the right caudate nucleus, and the right anterior cingulate cortex (ACC) in the PD-A group was compared with that in the PD-NA group. Further, the altered FC between the left cerebellum and the right cerebellum was significantly associated with anxiety symptoms in the PD-A group.

**Conclusion:**

The present study found abnormal regional cerebellum function as well as disruptions in the connectivity network within the cerebellum, caudate, and ACC in patients with PD-A. In addition, the FC between the left cerebellum and the right cerebellum was associated with anxiety symptoms in patients with PD. The present study indicated that cerebellar functional damage may be associated with anxiety symptoms in PD patients.

## Abbreviations

ACC: anterior cingulate cortex
ANOVA: one-way analysis of variance
FC: functional connectivity
GRF: Gaussian random field
HAMA: Hamilton anxiety rating scale
HAMD: Hamilton depression rating scale
H&Y: Hoehn & Yahr
MMSE: mini-mental state examination
MoCA: montreal cognitive assessment
MRI: magnetic resonance imaging
NCs: normal controls
NMS: non-motor symptom
NMSS: non-motor symptoms scale
PD: Parkinson’s disease
PD-A: PD patients with anxiety symptoms
PD-NA: PD patients without anxiety symptoms
ReHo: regional homogeneity
rs-fMRI: rest-state functional magnetic resonance imaging
ROI: region of interest
SPSS: statistical product and service solutions
UPDRS-III: unified PD rating scale part III
VMHC: voxel mirror homotopy connectivity

## 1 Introduction

Parkinson’s disease (PD), the second most prominent chronic progressive neurodegenerative disease in middle-aged and elderly people [1], has anxiety as one of its common neuropsychiatric symptoms. Studies have shown that up to 25–50% of the PD patients have varying degrees of anxiety [2]. It aggravates other motor symptoms, adversely affects the patient’s daily functioning and disease prognosis, reduces the patient’s quality of life, and increases the burden on the caregiver [3]. However, its insidious clinical symptoms make PD with anxiety difficult to identify and diagnose, and it has not received enough attention from researchers. Current neuromodulation system studies have suggested that striatum dopaminergic uptake levels [4], noradrenergic transmission [5], and serotonergic neuron systemic dysfunction [6] may be involved in the occurrence of PD with anxiety. Studies using magnetic resonance imaging (MRI) have found certain functional and structural changes in the cortical and subcortical areas of the brain of PD patients with anxiety, such as the gyrus return [7], the limbic system [8], and the cerebellum [9]. The neuronal circuit wiring plays a parallel role in anxiety symptoms in PD, in which the pathophysiological mechanism still has a large blind spot [10]. Therefore, characterizing the underlying neural mechanisms of patients with PD with anxiety symptoms using neuroimaging methods is urgent and imperative.

In PD patients, alteration of neuronal circuits such as subcortex-to-cortex pathways during neurodegeneration may explain the neuromechanism of anxiety symptoms [11]. Awareness has been increasing of a role for the cerebellum in affective behavior [12]; cerebellar dysfunction has been linked to motor symptoms in PD as well as to non-motor function, including anxiety symptoms. It is thus of direct interest to examine the connectivity within cerebellum and cortex to uncover neuroimaging evidence representative of anxiety symptoms in PD patients. This study’s aim was to explore aberrant functional connectivity (FC) throughout the cerebellar efferent pathways that project to the cerebral cortex using regional homogeneity (ReHo) and FC analysis among PD patients with anxiety symptoms.

## 2 Materials and methods

### 2.1 Participants

PD patients not taking anti-Parkinsonian drugs were recruited from the Department of Neurology, Affiliated Hospital of North Sichuan Medical College, between January 2019 and December 2020, and the diagnosis of PD followed the International Parkinson and Movement Disorder Society diagnostic criteria of 2015 for idiopathic PD [13]. The severity of anxiety symptoms was estimated using the Hamilton anxiety scale (HAMA). All PD patients with anxiety symptoms were diagnosed by HAMA scores higher than 12 [14], and all patients were separated into groups for PD patients with anxiety symptoms (PD-A) and PD patients without anxiety symptoms (PD-NA) based on this cutoff value. Furthermore, we followed up all participants for at least a year for diagnosis confirmation of PD. Patients meeting the following criteria were excluded: (a) progressive supranuclear paralysis, multiple system atrophy, vascular Parkinson’s syndrome, multiple system atrophy, and vascular Parkinson’s syndrome secondary parkinsonism; (b) MRI contraindications, such as claustrophobia, metal implants, and brain pathology or motor artifacts on MRI; and (c) depression symptoms with a score of ≥14 as per the Hamilton Depression Rating Scale (HAMD). Meanwhile, 30 age- and gender-matched volunteers with comparable levels of education were also recruited as normal controls (NCs).


**Informed consent:** Informed consent has been obtained from all individuals included in this study.
**Ethical approval:** The research related to human use has been complied with all the relevant national regulations, institutional policies, and in accordance with the tenets of the Helsinki Declaration, and has been approved by the ethics committee of the Affiliated Hospital of North Sichuan Medical College (No. 2019ER(R)016).

### 2.2 Clinical assessment

All drug-naïve PD patients were assessed using the non-motor symptom scale (NMSS), unified PD rating scale part III (UPDRS-III), and Hoehn & Yahr (H&Y) stage to access non-motor symptons (NMSs) and the motor symptoms. The montreal cognitive assessment (MoCA) was used to assess global cognitive function.

### 2.3 MRI data acquisition

All participants underwent MRI in a General Electric Discovery MR 750 3.0 Tesla MRI scanner with an orthonormal head coil with routine scanning to exclude severe brain tissue injury and motor artifacts of the brain. The echo-planar imaging sequence was used to acquire the resting-state blood-oxygen-level-dependent signal; the scanning parameters were as follows: echo time = 40 ms, repetition time = 2,000 ms, matrix = 64 × 64, flip angle = 90°, field of view = 24 cm × 24 cm, scanning time = 400 s, and image thickness space = 2 mm. A total of 200 volumes (33 slices/volume) were collected during rs-fMRI collection, resulting in 6,400 images for each subject.

During the whole resting-state scanning process, participants were instructed to close their eyes, keep their heads motionless, remain awake and relaxed, and minimize thinking activities. Meanwhile, cushions and ear plugs were used to prevent head movement and provide noise isolation.

### 2.4 Processing of rs-fMRI data

#### 2.4.1 Data preprocessing

SPM12 (http://www.mathworks.com) and RESTplus (http://restfmri.net/forum) software, which are based on the MATLAB R2013b platform (http://www.mathworks.com), were used to preprocess the rs-fMRI data [15]. Considering the stability of the magnetic field and the need for subjects to adapt to the environment, the first five volumes were removed to eliminate the influence of unstable signals. The remaining 195 volumes were corrected by the following preprocessed procedures: slice timing correction, realignment for head motion correction, and spatial normalization on the non-linear Montreal Neurological Institute-152 template using a 12-parameter affine transformation (resliced with the voxel size 3 mm^3^ × 3 mm^3^ × 3 mm^3^). Moreover, linear detrending, filtering (0.01–0.08 Hz), and nuisance covariate removal (white matter, cerebrospinal fluid, and 6-head motion parameters) were added to noise reduction. In the present study, any subject whose translation or rotation of head motion exceeded 2 mm or 2° was excluded from the following steps.

#### 2.4.2 ReHo analysis

Kendall’s coefficient of concordance (KCC) [16] was calculated for the preprocessed image to obtain the ReHo map of each subject. KCC was calculated by the consistency of the time series change between each voxel and its adjacent 26 voxels. Then, the standardized ReHo map was based on the ReHo value of each voxel divided by the mean ReHo value of the whole brain. ReHo diagrams of PD-A, PD-NA, and NC groups smoothed with a Gaussian filter of 4 mm of full width at half maximum will be used for subsequent statistical analysis.

#### 2.4.3 FC analysis

The FC analysis was implemented mainly by the CONN software [17]. Based on the ReHo results, the cerebellum with the maximum peak values was selected as the FC region of interest (ROI). We used seed-based FC analysis to extract the time series of each voxel in the ROI of each subject and calculate the mean ROI values [18], which were then used to compute the correlation with the time series of each voxel in the whole brain. We performed Fisher’s Z-transformation on the correlation coefficient, which helped us obtain the individual *Z*-score maps of each subject and ROI for subsequent statistical analysis.

### 2.5 Statistical analysis

All clinical data analyses were performed using the statistical package for the Social Sciences version 22.0 for Windows. Further, all continuous data were shown as the mean values ± standard deviation, and discontinuous data were shown as percentages. Moreover, we used Student’s *t*-test and one-way analysis of variance (ANOVA) to compare continuous data and the chi-square test to compare categorical variables between groups.

One-way ANOVA was used for evaluating the effect of anxiety on ReHo and FC among PD-A, PD-NA, and NC groups. Then, a two-sample *t*-test was performed for the *post hoc t* comparison-independent samples for comparing the differences between each pair of the three groups. Statistical significance was set at voxel level *P* < 0.001 and cluster level *P* < 0.05 based on Gaussian random field (GRF) correction for one-way ANOVA comparisons.

The correlation coefficients between clinical data comprising HAMA scores, NMSS, UPDRS-III, and ReHo values, as well as FC values of the inter-brain region, were evaluated using partial correlation analyses. Analyses were adjusted for age, gender, MoCA scores, and HAMD scores. All statistical tests were two-tailed; *P*-values <0.05 were considered statistically significant.

## 3 Results

### 3.1 Demographic and clinical data analysis

In this study, 50 newly diagnosed drug- naïve PD patients and 30 gender-, age- and education-level-matched NCs were finally included in this study. Anxiety symptoms were assessed according to the HAMA Scale, including 26 PD-A patients (HAMA >12 points) and 24 PD-NA patients (HAMA <12 points). Table 1 presents detailed demographic and clinical data for subjects. None of the subjects showed significant cognitive impairment or severe depression symptoms. The total HAMA score of the PD-A patients was significantly higher than that of the PD-NA patients (*P* < 0.001). Age of onset, course of disease, H&Y grading, UPDRS III, Mini-Mental State Examination (MMSE), MoCA, NMSS, and HAMD scores did not differ significantly between the two PD subgroups. There were no statistically significant differences between PD patients and NCs in terms of demographic variables.

**Table 1:** Comparison of demographic and clinical data of study subgroups

	NCs (*n* = 30)	PD (*n* = 50)	PD-A (*n* = 26)	PD-NA (*n* = 24)	PD vs NCs *T-*value (*P-*value)	PD-A vs PD-NA *T*-value (*P*-value)
Gender (M/F)	20/10	23/27	12/14	11/13	2.088(0.829)	2.147(0.764)
Age (years)	56.64 ± 7.83	60.43 ± 7.47	61.53 ± 9.87	58.47 ± 10.28	1.258(0.246)	1.248(0.304)
Education (years)	4.38 ± 2.56	4.98 ± 7.43	5.64 ± 9.28	3.73 ± 3.81	6.367(0.083)	5.682(0.069)
Onset age	—	—	59.35 ± 10.24	57.30 ± 10.53	—	0.936(0.354)
Duration (years)	—	—	1.28 ± 0.71	1.11 ± 0.67	—	0.824(0.424)
H & Y stage	—	—	1.3 ± 0.7	0.8 ± 0.8	—	1.943(0.058)
UPDRS III	—	—	22.39 ± 12.84	17.74 ± 9.93	—	3.146(0.078)
MMSE	27.92 ± 1.73	28.37 ± 1.58	28.13 ± 1.76	27.92 ± 2.78	1.437(0.245)	1.298(0.312)
MoCA	—	—	20.09 ± 4.54	18.88 ± 5.92	—	0.785(0.437)
NMSS	—	—	22.13 ± 58.79	21.25 ± 28.18	—	3.210(0.147)
HAMA	3.16 ± 1.89	—	17.91 ± 4.36	6.67 ± 3.52	—	9.692(<0.001^*^)
HAMD	2.97 ± 0.95	—	3.19 ± 1.27	3.75 ± 1.13	—	5.425(0.316)

PD-A, Parkinson’s Disease patients with anxiety; PD-NA, Parkinson’s Disease without anxiety; NCs, normal controls; UPDRS, the Unified Parkinson’s Disease Rating Scale; H&Y, Hoehn & Yahr; MoCA, the Montreal Cognitive Assessment; MMSE, Mini-Mental State Examination; NMSS, the Non-Motor Symptoms Scale; HAMA, the Hamilton Anxiety Rating Scale; HAMD, the Hamilton Depression Rating Scale.

^*^ *P* < 0.001 difference between PD-A and PD-NA.

### 3.2 ReHo analysis

**Figure 1:**
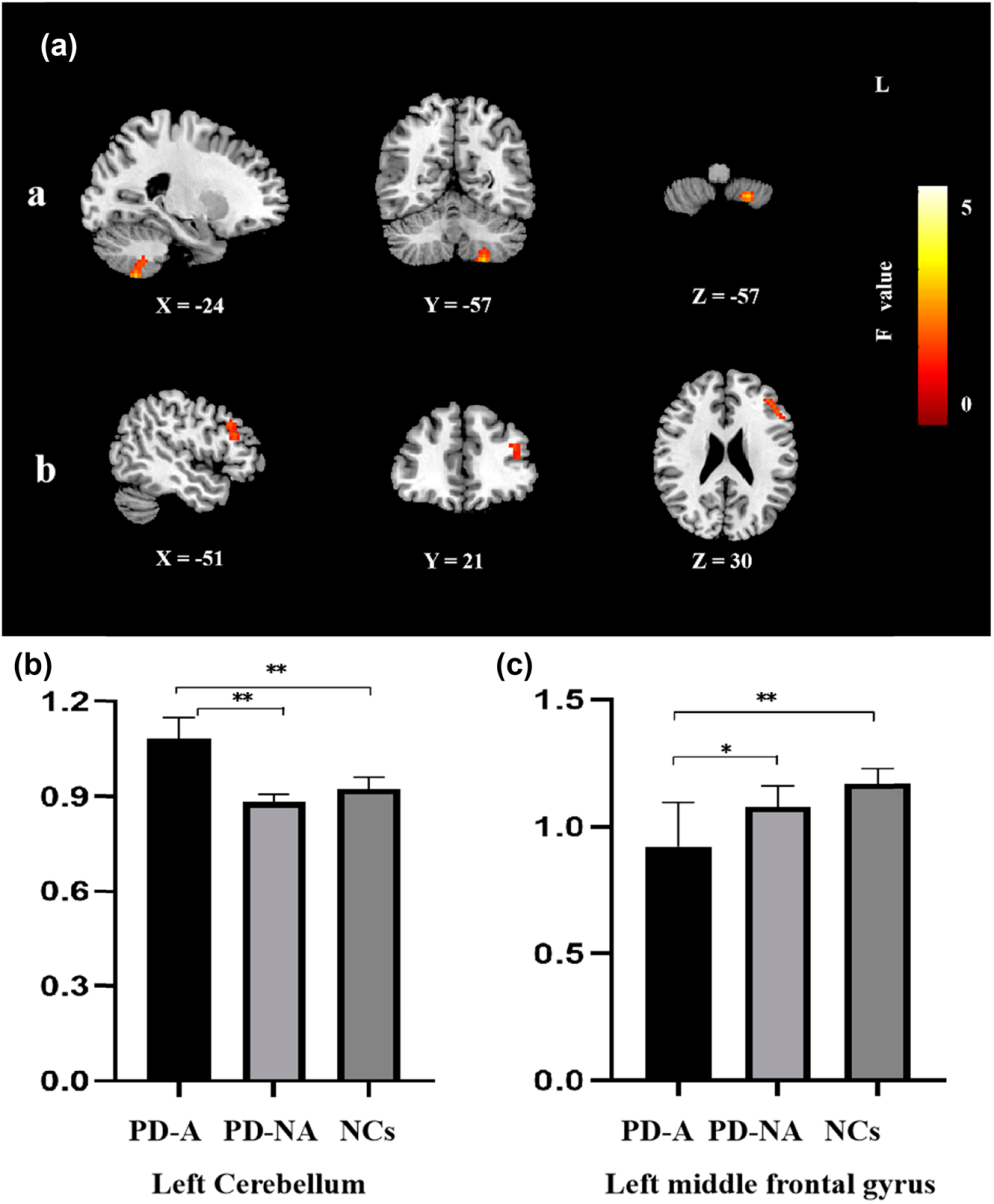
ReHo changes based on one-way ANOVA (a) and the *post hoc t* comparison-independent samples (b and c). (a) (Column demonstrates regions with significantly different ReHo values showing left cerebellum (a) and the left medial frontal gyrus (b) among the PD-A, PD-NA, and NC groups). The images shown here from left to right are sagittal, coronal, and axial views, respectively. (b) Column demonstrates ReHo value comparison of left cerebellum between *post hoc t* comparison of each pair among the three groups. (c) Column demonstrates ReHo value comparison of left middle frontal gyrus between *post hoc t* comparison of each pair among the three groups. Red represented increased ReHo. L indicates the left side. The color bar shows *F* value of one-way ANOVA analysis. Statistical significance was set at voxel level *P* < 0.001 and cluster level *P* < 0.05 based on GRF correction. ^*^ *P* < 0.05 and ^**^ *P* < 0.001.

The ANOVA showed that ReHo values of the left medial frontal gyrus and left cerebellum differed significantly among PD-A patients, PD-NA patients, and NCs (voxel level *P* < 0.001, cluster level *P* < 0.05, and GRF corrected). Further *post hoc t* comparison of each pair among the three groups showed that, compared with the PD-NA patients as well as the NCs, the PD-A patients had significantly decreased ReHo values in the left medial frontal gyrus and significantly increased ReHo values in the left cerebellum (Table 2, Figure 1).

**Table 2:** Comparison of ReHo differences among each pair among PD-A, PD-NA, and NCs

Anatomical region		Cluster size	MNI coordinates			Peak value (*T* value)
			*X*	*Y*	*Z*	
PD-A vs PD-NA	Left Cerebellum	23	−27	−55	−54	4.166
	Left middle frontal gyrus	15	−47	35	21	−2.655
						
PD-A vs NCs	Left Cerebellum	29	−22	−61	−51	3.372
	Left middle frontal gyrus	18	−52	25	34	−4.852

PD-A, Parkinson’s Disease patients with anxiety; PD-NA, Parkinson’s Disease without anxiety; NCs, Normal controls. The positive or negative of *T* values represent increases and decreases in ReHo values.

### 3.3 FC analysis

**Figure 2:**
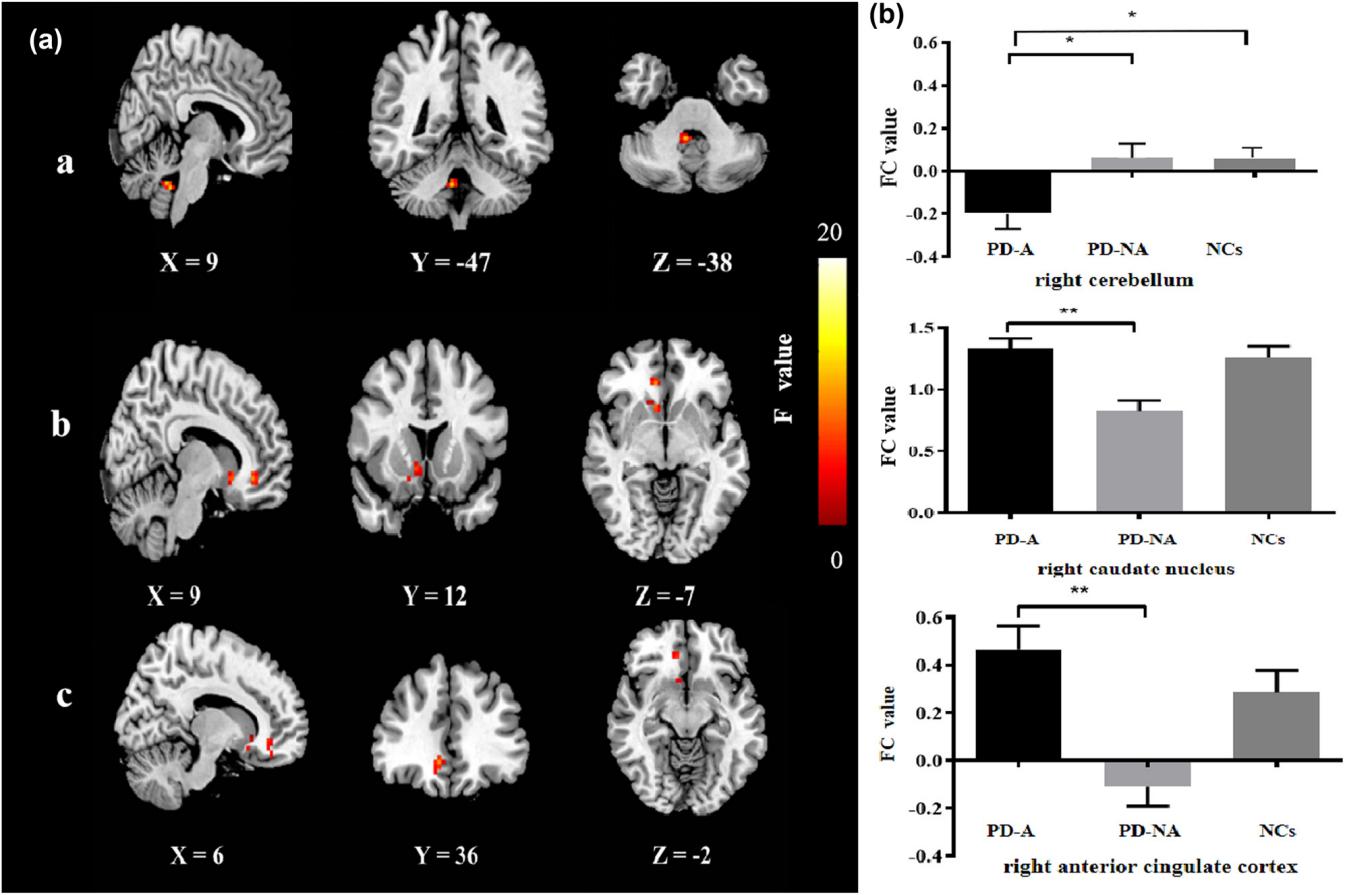
FC changes based on one-way ANOVA (a) and the *post hoc t* comparison-independent samples (b). (a) (Column demonstrates regions with significantly different FC with the left cerebellum showing right cerebellum (a), right caudate nucleus (b), and right ACC (c) among the PD-A, PD-NA, and NC groups). The image from left to right shows sagittal, coronal, and axial views, respectively. (b) Column demonstrates FC value comparison between *post hoc t* comparison of each pair among the three groups. Warm represented increase. L indicates the left side. The color bar shows *F* value of one-way ANOVA analysis. Statistical significance was set at voxel level *P* < 0.001 and cluster level *P* < 0.05 based on GRF correction. ^*^ *P* < 0.05 and ^**^ *P* < 0.001.

The left cerebellum, showing the maximum peak value of the ReHo changes, was selected as the ROI to research FC with other brain regions. The ANOVA revealed significant differences between the left cerebellum’s FC and that of the right cerebellum, right caudate nucleus, and right anterior cingulate cortex (ACC) among PD-A patients, PD-NA patients, and NCs (voxel level *P* < 0.001, cluster level *P* < 0.05, and GRF corrected). In *post hoc t* comparisons, PD-A FC values between the left and right cerebellum were decreased, whereas FC values between the left cerebellum and the right caudate nucleus and right ACC were increased compared with PD-NA FC values. Compared with NCs, the FC values between left and right cerebellum in PD-A were significantly lower (voxel level *P* < 0.001, cluster level *P* < 0.05, and GRF corrected) (Table 3, Figure 2).

**Table 3:** Comparison of FC values among each pair among PD-A, PD-NA and NCs

Anatomical region		Cluster size	MNI coordinates			Peak value (*T* value)
			*X*	*Y*	*Z*	
PD-A vs PD-NA	Right cerebellum	15	6	−50	−32	−4.251
	Right caudate nucleus	16	15	18	−15	4.267
	Right ACC	12	8	37	−8	4.158
PD-A vs NCs	Right cerebellum	14	5	−47	−35	−4.506

PD-A, Parkinson’s disease patients with anxiety; PD-NA, Parkinson’s disease without anxiety; NCs, normal controls.

### 3.4 Partial correlation analysis

**Figure 3:**
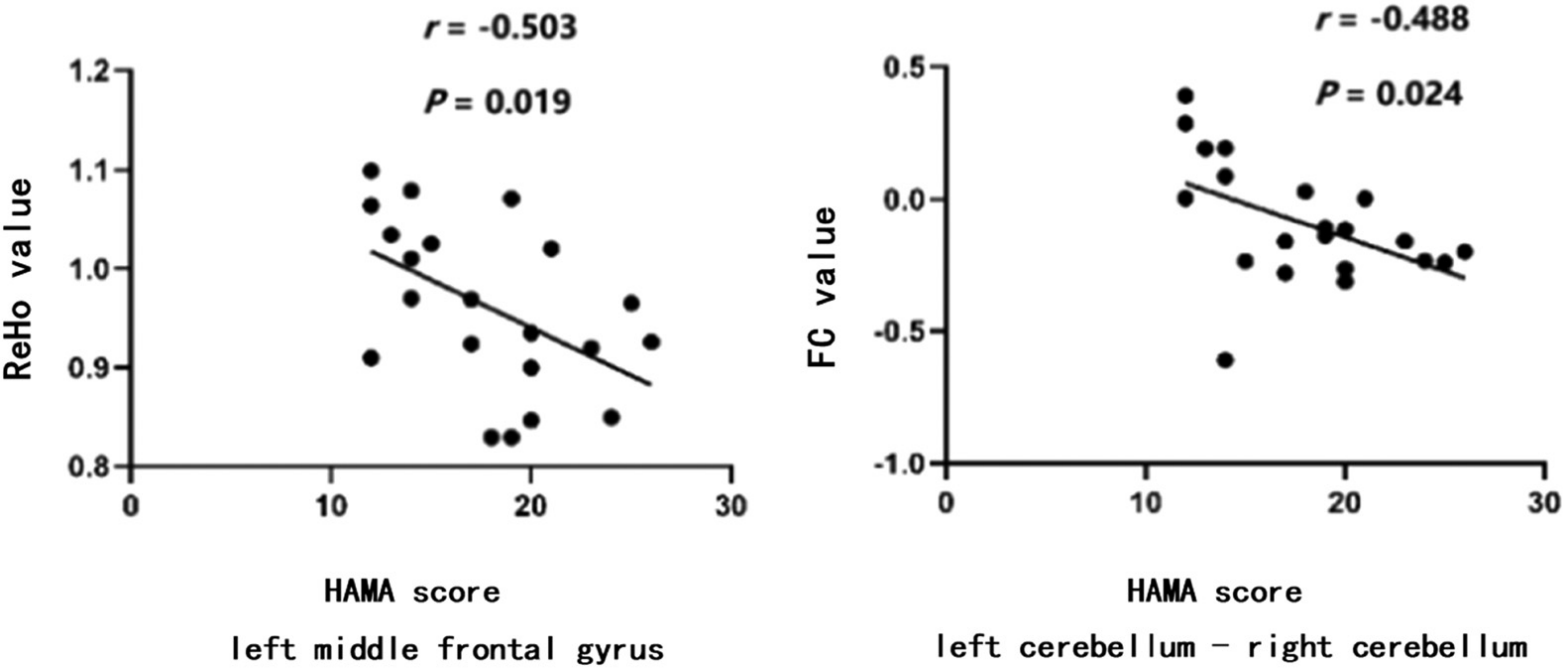
Partial correlation analysis in the PD-A between imaging data and HAMA scores.

The partial correlation analysis revealed a negative correlation between HAMA scores and ReHo values in the left middle frontal gyrus of the PD-A patients (*r* = −0.503 and *P* = 0.019). FC values between the left and right cerebellum showed a significantly negative correlation with HAMA scores of PD-A patients (*r* = −0.488 and *P* = 0.024) (Figure 3). However, there was no significant correlation between UPDRS-III score, NMSS score, and imaging data (ReHo and FC) of altered brain areas in PD-A patients when correcting for age, gender, MMSE, and HAMD scores.

## 4 Discussion

The present study investigated the neuroimaging mechanism of changes in neuronal functional activities and functional connections on newly diagnosed drug-naïve PD patients with anxiety symptoms based on ReHo and ROI-based FC analysis. Consequently, we found that ReHo values in the left cerebellum were significantly increased, while it was significantly decreased in the left middle frontal gyrus. In addition, the FC values of the left and right cerebellum were significantly decreased, while the FC of the left cerebellum and right caudate nucleus as well as right ACC were higher in PD patients with anxiety than in PD patients without anxiety. The *partial* correlation analysis demonstrated that significant anxiety symptoms in PD patients were negatively correlated with ReHo values in the left middle frontal gyrus and decreased FC values within the left and right cerebellum. This suggests that the underlying neuropathologic mechanism of PD with anxiety was related to the dysfunction of the local cerebellum network.

In this study, it was found that the ReHo value of the left medial frontal gyrus was reduced and that of the left cerebellum was significantly increased. The left medial frontal gyrus has a greater top-down loop regulation function [19] in the PD-A group than in the PD-NA group. This suggests that the neural circuits between the cerebellum and medial frontal gyrus were dysfunctional in the anxiety state. The middle frontal gyrus participates in advanced brain functions such as emotion, memory, and thinking and often plays an instrumental role in emotional control [20]. Neuroimaging studies suggest the involvement of a similar neural circuit in the development of anxiety disorders: the frontal-subcortical and the cortical-marginal pathway [19,21]. In addition, the reduction in prefrontal lobe activity has been observed in patients with anxiety, which through top-down attenuation inhibits cortical and subcortical structures, leading to emotional regulation imbalance, abnormalities in processing negative emotions such as anxiety and fear, and the occurrence of anxiety [19]. Reduction in ventral activity in the prefrontal cortex and enhancement of dorsal activity were reportedly associated with anxiety, suggesting that various subregions of the prefrontal cortex play different roles in anxiety [22]. However, the specific mechanism by which the cerebellum regulates anxiety through the middle frontal gyrus is not yet clear.

Our findings demonstrated that the spontaneous neuronal activity of the cerebellum was disrupted in PD patients with anxiety symptoms. It is well-known that the cerebellum participates in motor function; however, previous studies have confirmed that the cerebellum is closely associated with the cortical and subcortical brain regions associated with anxiety, such as the amygdala [23], prefrontal cortex [24], and cingulate gyrus [25]. Structural MRI studies found that the cerebellar tonsils and cerebellar right lobes in PD patients with anxiety had significantly reduced gray matter volume compared with those of healthy controls [26]. The cerebellum may be an important component of the neural network of anxiety [27]. The existing research suggests that the cerebellum not only participates in the regulation of muscle tension while coordinating autonomous activities but also finely adjusts the process of sensory information acquisition, which has been widely recognized. Our results showed that the ReHo value of the left cerebellum increased in the PD-A patient group, suggesting that the cerebellum is an important anatomical structure in anxiety regulation.

The results – increased connectivity within left cerebellum and right caudate nucleus as well as right ACC and decreased connectivity between the left cerebellum and right cerebellum – also suggested that there is imbalance in the local neural brain functional network of PD patients with anxiety symptoms and that the cerebellum may be an important component of the neural network of anxiety.

The ACC is involved in the formation of emotional networks and in the integration of negative emotions [28]. Whalen et al. found that the ACC is significantly activated after stimulating the emotion of disgust in generalized anxiety patients, which was related to a good treatment prognosis in anxiety patients with high ACC activity [29]. This is consistent with our study’s finding for PD patients with anxiety symptoms. Further speculation is that the ACC perceives involuntary nervous and tremor motility based on abnormal activities in the cerebellum, thereby strengthening the functional network connection with the cerebellum so as to regulate anxiety symptoms. However, both the specific mechanism of FC changes in the cerebellum and the involvement of ACC in brain functional networks with regard to PD anxiety symptoms need further study.

Similarly, we found enhanced FC between the left cerebellum and right caudate nucleus in PD-A patients, but no similar changes in functional connections were reported in the previous study. Nevertheless, it is well-known that as subcortical structures, the cerebellum and striatum affect motor symptoms and emotional dysfunction [30]. Previous research has found that the density of dopamine transporters was increased in the caudate nucleus of PD-A patients, which was significantly positively correlated with the severity of anxiety symptoms [31]. It is confirmed that the substantia nigra striatum pathway was related to PD anxiety symptoms. Thus, as the fundings in the study, in PD-A patients, we speculated that the abnormal functional connections of the cerebellum and caudate nucleus were related to the impaired integrity of the cerebellar–striatum nerve loop in PD patients with anxiety symptoms.

Similarly, we found the presence of decreased FC between left and right cerebellum in PD-A patients, but not in NCs and PD-NA patients and that it was negatively correlated with HAMA score. Voxel mirror homotopy connectivity (VMHC) analysis found that bilateral cerebellum VMHC value decreased and was negatively correlated with HAMA scores in patients with generalized anxiety disorder [32]. Overall, our finding suggests that PD anxiety may be associated with decreased synchronization of bilateral cerebellar functions. Geometrically isotopic brain region connectivity decreases as anxiety severity increases, and abnormalities in the mirror bilateral cerebellar functional connection network may be involved in the brain functional network mechanism of PD anxiety symptoms.

We all know that the changes in the functional connections between the amygdala, prefrontal lobe, and cerebellum can be widely involved in regulating anxiety symptoms [33]. The present study did not find abnormal amygdala function in PD anxiety patients, which may be due to the mild degree of PD anxiety symptoms of patients recruited in our study; and the compensatory enhancement of left cerebellum functional activities in the early stage and the functional changes in the amygdala were inhibited. In addition, it is important to note that the H&Y grade and UPDRS III score had no significant relationship with left cerebellum function connection, which suggested that movement had little influence on early PD patients with anxiety. Abnormal neural networks in the cerebellum may be the result of anxiety symptoms rather than movement.

In conclusion, all patients included in the study were newly diagnosed, drug-naïve PD patients, thus effectively reducing drug bias in the outcome. Anxiety symptoms are less affected by the course of disease, severity, or exercise symptoms in the early stage of PD. In summary, our study shows that anxiety symptoms in PD patients are associated with changes in neural functional activity in the bilateral cerebellum, left middle frontal gyrus, right caudate nucleus, and right ACC.

## 5 Conclusion

There is abnormal spontaneous electrical activity in the left cerebellum and left middle frontal gyrus. Changes in the functional connections between the left and right cerebellum and in the left cerebellum, the right caudate nucleus, and the right ACC may represent underlying mechanisms of PD with anxiety symptoms and may thus be important potential biomarkers for PD with anxiety symptoms. Nonetheless, further clarification of long-term neural implications of anxiety symptoms in PD is needed.

## References

[1] Sauerbier A, Jenner P, Todorova A, Chaudhuri KR. Non motor subtypes and Parkinson’s disease. Parkinsonism Relat Disor. 2016;22(Suppl 1):S41–6. 10.1016/j.parkreldis.2015.09.027 26459660

[2] Broen MP, Narayen NE, Kuijf ML, Dissanayaka NN, Leentjens AF. Prevalence of anxiety in Parkinson’s disease: a systematic review and meta-analysis. Mov Disord. 2016;31(8):1125–33. 10.1002/mds.26643 27125963

[3] Dissanayaka NN, Sellbach A, Matheson S, O'Sullivan JD, Silburn PA, Byrne GJ, et al. Anxiety disorders in Parkinson’s disease: prevalence and risk factors. Mov Disord. 2010;25(7):838–45. 10.1002/mds.22833 20461800

[4] Moonen A, Weiss PH, Wiesing M, Weidner R, Fink GR, Reijnders J, et al. An fMRI study into emotional processing in Parkinson’s disease: does increased medial prefrontal activation compensate for striatal dysfunction? PLoS One. 2017;12(5):e0177085. 10.1371/journal.pone.0177085 PMC5423613 28486506

[5] Blonder LX, Slevin JT. Emotional dysfunction in Parkinson’s disease. Behav Neurol. 2011;24(3):201–17. 10.3233/BEN-2011-0329 PMC3177157 21876260

[6] Joling M, van den Heuvel OA, Berendse HW, Booij J, Vriend C. Serotonin transporter binding and anxiety symptoms in Parkinson’s disease. J Neurol Neurosurg Psychiatry. 2018;89(1):89–94. 10.1136/jnnp-2017-316193 28899958

[7] O'callaghan C, Shine JM, Lewis SJ, Hornberger M. Neuropsychiatric symptoms in Parkinson’s disease: fronto-striatal atrophy contributions. Parkinsonism Relat Disord. 2014;20(8):867–72. 10.1016/j.parkreldis.2014.04.027 24866458

[8] Wee N, Wen MC, Kandiah N, Chander RJ, Ng A, Au WL, et al. Neural correlates of anxiety symptoms in mild Parkinson’s disease: a prospective longitudinal voxel-based morphometry study. J Neurological Sci. 2016;371:131–6. 10.1016/j.jns.2016.10.021 27871434

[9] Wang X, Li J, Yuan Y, Wang M, Ding J, Zhang J, et al. Altered putamen functional connectivity is associated with anxiety disorder in Parkinson’s disease. Oncotarget. 2017;8(46):81377–86. 10.18632/oncotarget.18996 PMC5655292 29113397

[10] Carey G, Görmezoğlu M, de Jong JJA, Hofman P, Backes WH, Dujardin K, et al. Neuroimaging of anxiety in Parkinson’s disease: a systematic review. Mov Disord. 2021 Feb;36(2):327–39. 10.1002/mds.28404, Epub 2020 Dec 2. PMC7984351 33289195

[11] Thobois S, Prange S, Sgambato-Faure V, Tremblay L, Broussolle E. Imaging the etiology of apathy, anxiety, and depression in Parkinson’s disease: implication for treatment. Curr Neurol Neurosci Rep. 2017;17:76. 10.1007/s11910-017-0788-0 28822071

[12] Gill JS, Sillitoe RV. Functional outcomes of cerebellar malformations. Front Cell Neurosci. 2019 Oct 4;13:441. 10.3389/fncel.2019.00441 PMC6787289 31636540

[13] Postuma RB, Berg D, Stern M, Poewe W, Olanow CW, Oertel W, et al. MDS clinical diagnostic criteria for Parkinson’s disease. Mov Disord. 2015;30(12):1591–601. 10.1002/mds.26424 26474316

[14] Zimmerman M, Martin J, Clark H, McGonigal P, Harris L, Holst CG. Measuring anxiety in depressed patients: a comparison of the Hamilton anxiety rating scale and the DSM-5 anxious distress specifier interview. J Psychiatr Res. 2017;93:59–63. 10.1016/j.jpsychires.2017.05.014 28586699

[15] Chen JE, Glover GH. Functional magnetic resonance imaging methods. Neuropsychol Rev. 2015;25(3):289–313. 10.1007/s11065-015-9294-9 PMC4565730 26248581

[16] Zang Y, Jiang T, Lu Y, He Y, Tian L. Regional homogeneity approach to fMRI data analysis. NeuroImage. 2004;22(1):394–400. 10.1016/j.neuroimage.2003.12.030 15110032

[17] Fu Z, Tu Y, Di X, Biswal BB, Calhoun VD, Zhang Z. Associations between functional connectivity dynamics and BOLD dynamics are heterogeneous across brain networks. Front Hum Neurosci. 2017;11:593. 10.3389/fnhum.2017.00593 PMC5770626 29375335

[18] Woolrich MW, Ripley BD, Brady M, Smith SM. Temporal autocorrelation in univariate linear modeling of FMRI data. Neuroimage. 2001;14(6):1370–86. 10.1006/nimg.2001.0931 11707093

[19] Phan KL, Wager TD, Taylor SF, Liberzon I. Functional neuroimaging studies of human emotions. CNS Spectr. 2004;9(4):258–66. 10.1017/s1092852900009196 15048050

[20] Liang P, Deshpande G, Zhao S, Liu J, Hu X, Li K. Altered directional connectivity between emotion network and motor network in Parkinson’s disease with depression. Medicine. 2016;95(30):4222. 10.1097/MD.0000000000004222 PMC5265831 27472694

[21] Myers-Schulz B, Koenigs M. Functional anatomy of ventromedial prefrontal cortex: implications for mood and anxiety disorders. Mol Psychiatry. 2012;17(2):132–41. 10.1038/mp.2011.88 PMC3937071 21788943

[22] Straube T, Schmidt S, Weiss T, Mentzel HJ, Miltner WH. Dynamic activation of the anterior cingulate cortex during anticipatory anxiety. Neuroimage. 2009;44(3):975–81. 10.1016/j.neuroimage.2008.10.022 19027072

[23] Farley SJ, Radley JJ, Freeman JH. Amygdala modulation of cerebellar learning. J Neurosci. 2016;36(7):2190–201. 10.1523/JNEUROSCI.3361-15.2016 PMC4756154 26888929

[24] Watson TC, Becker N, Apps R, Jones MW. Back to front: cerebellar connections and interactions with the prefrontal cortex. Front Syst Neurosci. 2014;8:4. 10.3389/fnsys.2014.00004 PMC3912388 24550789

[25] Yamawaki S, Okada G, Okamoto Y, Liberzon I. Mood dysregulation and stabilization: perspectives from emotional cognitive neuroscience. Int J Neuropsychopharmacol. 2012;15(5):681–94. 10.1017/S1461145711000757 21733243

[26] Ma X, Su W, Li S, Li C, Wang R, Chen M, et al. Cerebellar atrophy in different subtypes of Parkinson’s disease. J Neurol Sci. 2018;392:105–12. 10.1016/j.jns.2018.06.027 30036781

[27] Otsuka S, Konno K, Abe M, Motohashi J, Kohda K, Sakimura K, et al. Roles of Cbln1 in non-motor functions of mice. J Neurosci. 2016;36(46):11801–16. 10.1523/JNEUROSCI.0322-16.2016 PMC6705638 27852787

[28] Gasquoine PG. Localization of function in anterior cingulate cortex: from psychosurgery to functional neuroimaging. Neurosci Biobehav Rev. 2013;37(3):340–8. 10.1016/j.neubiorev.2013.01.002 23313645

[29] Whalen PJ, Johnstone T, Somerville LH, Nitschke JB, Polis S, Alexander AL, et al. A functional magnetic resonance. Imaging predictor of treatment response to venlafaxine in generalized anxiety disorder. Biol Psychiatry. 2008;63(9):858–63. 10.1016/j.biopsych.2007.08.019 PMC2654286 17964548

[30] Strick PL, Dum RP, Fiez JA. Cerebellum and nonmotor function. Annu Rev Neurosci. 2009;32:413–34. 10.1146/annurev.neuro.31.060407.125606 19555291

[31] Moriyama TS, Felicio AC, Chagas MH, Tardelli VS, Ferraz HB, Tumas V, et al. Increased dopamine transporter density in Parkinson’s disease patients with social anxiety disorder. J Neurol Sci. 2011;310(1–2):53–7. 10.1016/j.jns.2011.06.056 21783205

[32] van der Velden RMJ, Broen MPG, Kuijf ML, Leentjens A. Frequency of mood and anxiety fluctuations in Parkinson’s disease patients with motor fluctuations: a systematic review. Mov Disord. 2018;33(10):1521–7. 10.1002/mds.27465 30225905

[33] Etkin A, Prater KE, Schatzberg AF, Menon V, Greicius MD. Disrupted amygdalar subregion functional connectivity and evidence of a compensatory network in generalized anxiety disorder. Arch Gen Psychiatry. 2009;66(12):1361–72. 10.1001/archgenpsychiatry.2009.104 PMC12553334 19996041

